# Pancreatic lipoma: a pancreatic incidentaloma; diagnosis with ultrasound, computed tomography and magnetic resonance imaging

**DOI:** 10.1259/bjrcr.20150507

**Published:** 2016-11-02

**Authors:** Suhas Aithal Sitharama, Manju Bashini, Kannan Gunasekaran, Deepak Barathi Subramania

**Affiliations:** Department of Radiodiagnosis, Jawaharlal Institute of Postgraduate Medical Education and Research, Puducherry, India

## Abstract

Pancreatic lipomas are rare. We present a case of incidentally discovered pancreatic lipoma in a 45-year-old female suffering from metastatic ovarian carcinoma who was referred to radiology for follow-up imaging. Fat-containing tumours originating from the pancreas are very rare. Most lipomasshow characteristic features on imaging that allow their differentiation. In most cases, accurate diagnosis is attained without any histopathological confirmation. We present the imaging features of pancreatic lipoma on ultrasound, CT scan and MRI, the differential diagnosis and a brief review of the literature.

## Background

Pancreatic mesenchymal tumours account for 1–2% of all pancreatic tumours; they are classified according to their histologic origin.^1–3^ Rarer among them are those involving the fatty tissue (lipoma and liposarcoma). On ultrasound imaging, lipomas are usually hyperechoic, although some lesions may demonstrate hypoechogenicity. Routine use of imaging and familiarity of the radiologists with this condition will increase the number of cases of pancreatic lipomas being diagnosed.

## Case Presentation

The patient is a 45-year-old female who was referred to the radiology department from the regional cancer center for imaging evaluation of a sonographically detected ovarian carcinoma. She was asymptomatic for the pancreatic lesion. She underwent CT imaging as a part of routine follow-up, which identified a pancreatic lipoma. Ultrasound and MRI were performed subsequently. MRI corroborated the CT scan finding.

**Figure 1.**
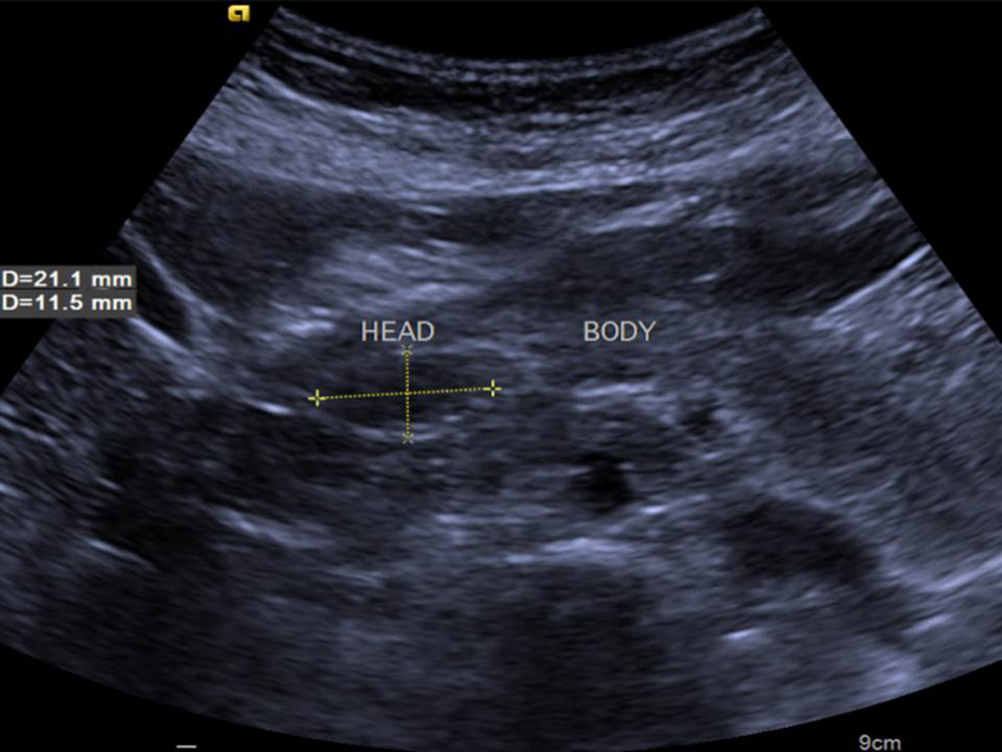
Transverse grayscale (B-mode) ultrasound image showing hypo- to isoechoic lesion on the head of the pancreas.

On ultrasound (Figure 1), the lesion was iso to hypoechoic when compared with liver echogenicity and located on the head of the pancreas. It appeared as a soft lesion on the elastographic grayscale image. The rest of the pancreas was normal in size and echogenicity, without significant dilation of the main pancreatic duct.

**Figure 2.**
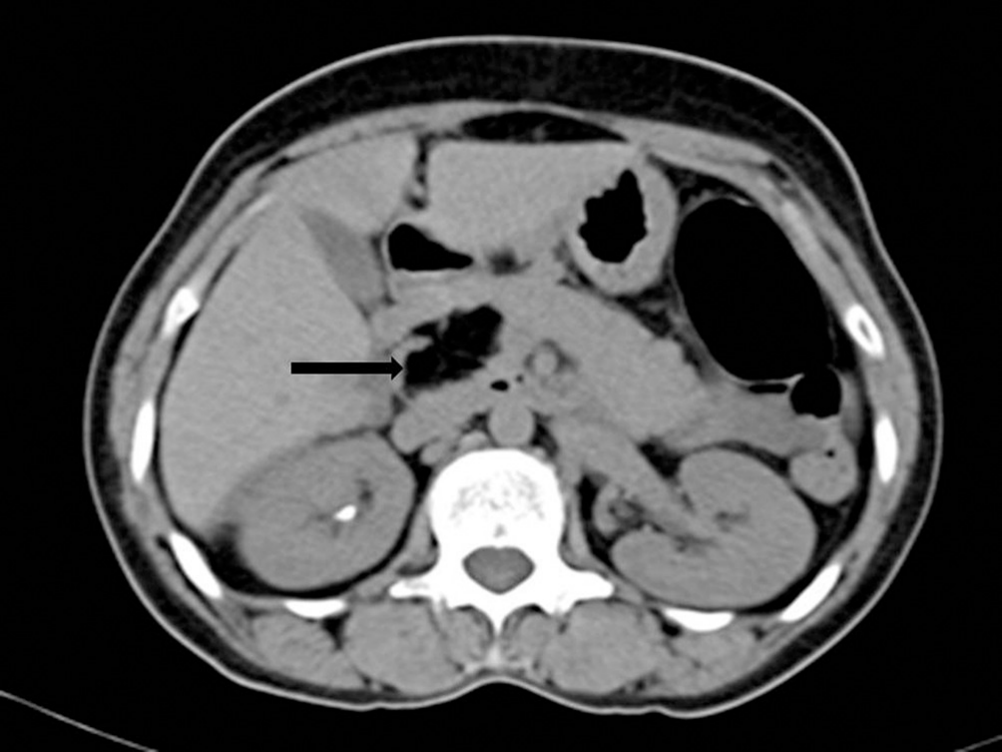
Non-contrast CT scan shows a well-circumscribed focal lesion (arrow) on the pancreatic head, measuring 35 x 35 x 19 mm and with a density of −106 Hounsfield units, consistent with fatty tissue.

**Figure 3.**
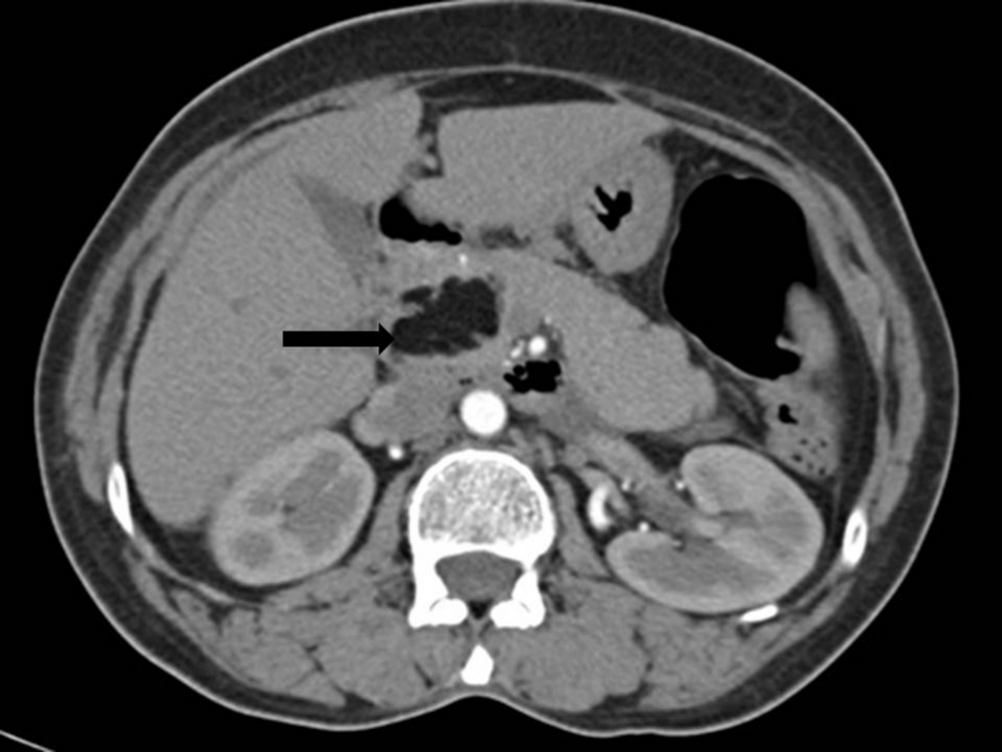
Contrast-enhanced CT scan shows a homogeneous focal mass (arrow) on the pancreatic head. The mass was isodense with fatty tissue and interlobular septa, and without central or peripheral contrast enhancement (arrow).

Plain and contrast sections of the CT scan of the abdomen (Figures 2 and 3) showed bilateral enhancing adnexal lesions; a well-defined, lobulated, homogeneous fat density lesion of approximately 3.5 cm (transverse) × 1.9 cm (anteroposterior) × 3.5 cm (craniocaudal length) on the head of the pancreas without infiltration of peripancreatic fatty tissue; and widening of the pancreatic duct and common bile duct.

**Figure 4.**
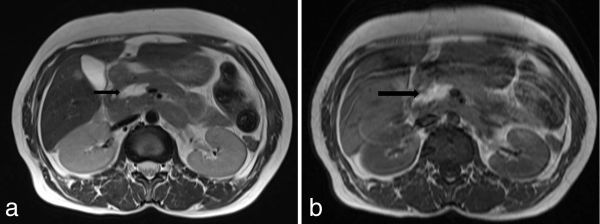
(a) Axial *T*_2 _and (b) *T*_1_ weighted MRI showing a hyperintense lobulated mass (arrows) on the head of the pancreas.

**Figure 5.**
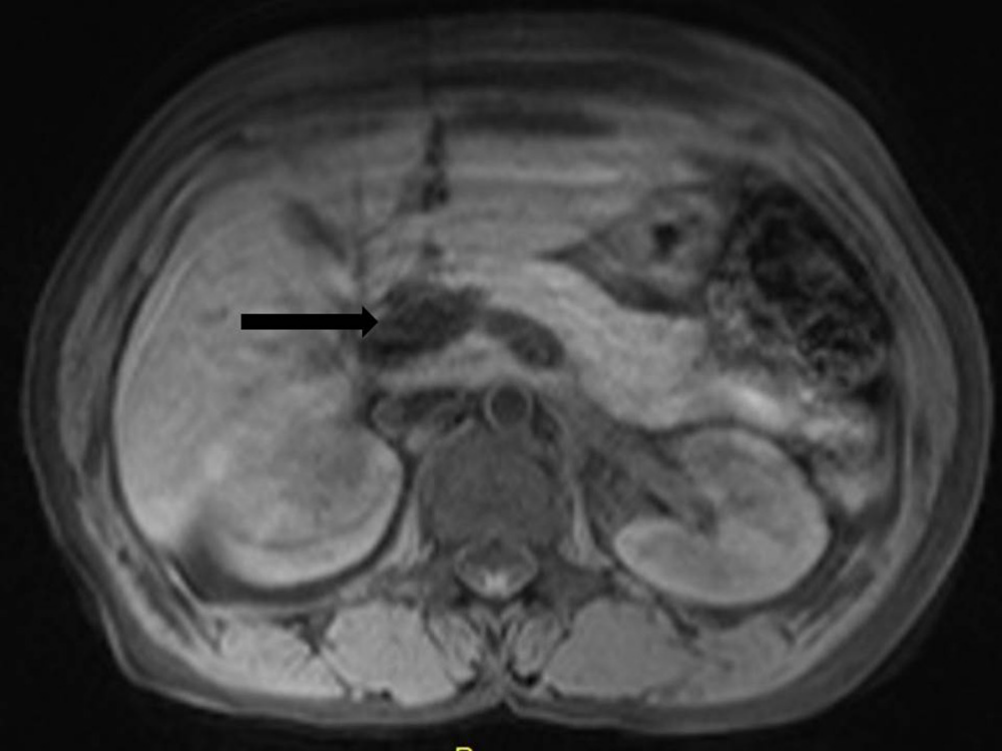
Axial *T*_1_ weighted fat-suppressed sequence showing suppression of *T*_1_ hyperintensity (black arrow) within the lesion, suggesting a lesion of fatty nature.

MRI of the abdomen was performed to confirm its benignity, as it was a leave-alone lesion. *T_2_* (Figure 4a), *T_1_* (Figure 4b) and *T*_1_ fat-suppressed images (Figure 5) were taken. It was hyperintense on *T*_1_ and *T*_2 _images. *T*_1_ hyperintensity was suppressed on fat-suppressed sequences, confirming the fatty nature of the lesion.

The patient was being followed up for ovarian cancer. Prior CT scans had already revealed the pancreatic lesion and when compared with the recent scan, the size of the lesion appeared stable, suggesting benignity. Therefore, histological confirmation was not obtained.

## Discussion

Bigard et al^4^ were the first to describe a pancreatic lipoma in 1989 as a hypoechoic mass on the head of the pancreas. Epithelial tumours constitute the majority of pancreatic tumours, including adenocarcinoma, which accounts for 85% of all pancreatic tumours. Among pancreatic tumours, nonductal tumours are uncommon and account for only about 5–15% of pancreatic neoplasms. Only 1–2% of pancreatic neoplasms are constituted by mesenchymal tumours.^5^

These non-ductal neoplasms may be benign (lymphangioma, lipoma, fibroma, neurofibroma, schwannoma, haemangioma, haemangioendothelioma, leiomyoma and desmoid tumours) or malignant (lymphoma, pancreatoblastoma, liposarcoma, fibrous histiocytoma, haemangiopericytoma and neuroectodermal neoplasms) and are identified histopathologically.^5^

Pancreatic lipomas are rare tumours. In 2008, Xu et al^6^ reviewed 169 cases of protruding lesions in the duodenum and found only one lipoma (0.59%). In 2006, Hois et al^7^ analyzed 6000 CT scans and identified pancreatic lipomas in 5 of them (0.083%). In 2013, Hammond et al^8^ also described a variety of uncommon benign and malignant pancreatic neoplasms. Recently, in 2014, Gossner et al^9^ retrospectively reviewed 100 abdominal CT scans, of which 6 had pancreatic lipomas (6%). Therefore, the incidence of lipomas may not be so rare as previously thought, but they may have been missed or not reported.

A pancreatic lipoma is a mass made up of mature adipose cells, which is encapsulated in a collagen layer that facilitates enucleation and helps differentiate lipoma from lipomatosis.^10^

Ultrasound cannot be the gold standard method for detecting pancreatic lipomas, as these can be hypo-, iso- or hyperechoic lesions. In our case, it was iso- to hypoechoic when compared with liver echogenicity.

CT scan is the most effective imaging modality for diagnosing pancreatic lipomas. Usually, a pancreatic lipoma is a homogeneously hypodense, non-enhancing mass with attenuation values ranging from −30 to −120 Hounsfield units. Infiltration into the surrounding tissues is unusual.^11,12^

Lipomas appear hyperintense on both *T*_1_ and *T*_2_ weighted sequences, similar to intra-abdominal and subcutaneous fat. Fat-suppressed, *T*_1_ weighted images show homogeneous suppression of signal intensity within the tumour.

Although they may be asymptomatic, some lipomas produce pancreatic or biliary obstruction or both. In our case, the patient had no symptoms related to the lesion.

Butler et al^13^ studied 74 cases of intrapancreatic lipomas for a period of 12 years. The majority of them were asymptomatic at presentation and none of them required intervention, or showed interval growth or change on imaging appearance. They concluded that these show stable size, morphology and benign course, and only required short-term interval observation for proving their stability and differentiating them from early liposarcoma.

Tumour size can range from 1 to 30 cm; in our case, it had a maximum dimension of 3.5 cm.

In most of the reported cases, the lesions were located on the head of the pancreas,^3–5,11,12,14–16^ as in our case. In only a few cases were lesions found on the tail^2,11,17,18^ and the body of the pancreas.^2,11^

Important differential diagnosis that can be considered include fatty replacement, pseudohypertrophic lipomatosis, liposarcoma and teratoma.

*Fatty replacement* can be focal or diffuse. The pancreas decreases in size (in the anteroposterior diameter) but with maintained lobulations. It may be associated with advanced age, obesity, Cushing syndrome or diabetes. The well-defined capsule of lipomas is absent in fatty replacement. *Pseudohypertrophic lipomatosis* is a rare condition where there is enlargement of the pancreas owing to increase in fat content and decrease in the number of constituent cells, especially exocrine tissue with intact islets of Langerhans. MRI shows total replacement of the pancreatic parenchyma by fat tissue. From a clinician’s point of view, differentiation between lipomas and focal fatty infiltration of the pancreas is not significant, as they do not require any intervention or treatment (especially lesions < 3 cm). *Teratoma* is rare in the pancreas, usually diagnosed when typical features of teratoma are seen. It is generally symptomatic and requires surgical treatment. *Liposarcoma* is an irregular mixed fat and soft tissue density lesion with patchy areas of enhancement. It is clinically important as it is the only fatty pancreatic lesion that requires surgery.

As present, imaging techniques are very accurate, and in most cases, there is no need for histopathological confirmation of pancreatic lipoma. It is needed in patients with larger tumours, where differentiation from malignant lipid-containing tumours becomes difficult. There was no histopathological confirmation obtained in our case as well.

## Learning points

1. Pancreatic lipoma is a rare non-ductal mesenchymal tumour that is being recognized more frequently nowadays.
2. CT scan is confirmative but MRI is the imaging modality to detect lesions of smaller size (< 3cm).
3. Pancreatic lipoma is commonly asymptomatic and incidentally detected.
4. Pancreatic lipoma is treated only if vital structures such as duodenum or ampulla are compressed or if any malignant changes occur.

## Consent

Informed consent was obtained from the patient for publication of this case report.
